# Supplementary material for: Patterns of Intron Gain and Loss in Fungi
Source: PLoS Biol. 2004 Nov 30;2(12):e422. doi: 10.1371/journal.pbio.0020422 (PMC532390; doi:10.1371/journal.pbio.0020422)
Supplement: Table S1 — Also available at http://genes.mit.edu/NielsenEtAl/. (4.3 MB ZIP). [file pbio.0020422.st001.zip › NielsenEtAl/html/99.html]

AN2965.1.NCU01269.1.MG06892.1.FG01908.1


```
 CLUSTAL W (1.82) Multiple Sequence Alignments - Introns Inserted


Sequence 1: NCU01269.1	597 aa
Sequence 2: MG06892.1	604 aa
Sequence 3: FG01908.1	598 aa
Sequence 4: AN2965.1	592 aa
Alignment Length: 618 aa
Number Identitical Residues: 320 aa
Alignment Score (without introns) 15475


MG06892.1 	MGVNGDAAVSLGLASSSKRTVRASNSPAVG---ARASTPPPAIDRNHFDGGNHRDSQGGR
NCU01269.1	MVTTGDVSGRAAHSAGTKRSSYDTRSQAVHPSTTRVTRSSRLQTPPPPSSGNSREEPPTE
FG01908.1 	-----------MNSQPTKRAIDESKSHTATP----RSSTPPRSDRTRLEPRYSLDNGPTR
AN2965.1  	-MSESNRASSNPAIYGSARALRSTGTHIVSP--------PGSRTPPSMPAKTTLYFPEPT
          	    .. :        : *:   : :  . .        .                    

MG06892.1 	RGRN--GARLSDASSHDLDLDAANHALRREVLSRQSRETTPNGSPHRKRQRITGDR2FIP
NCU01269.1	IARNPRAPTRTEDHVNSLDVEAVDSALRREIQQRQ---TSPGPSSHRKRQRINGDR2FIP
FG01908.1 	PARN-YGRKSEGARGSHLDIEAVDSALLREFQRPQ-RESTPGASPHRKRQRINGDR2FIP
AN2965.1  	GVHH-----TSRSVSGPIDPNALAKALREYEDAGRSRERTPGTSPSRKRQRVYGDR2FIP
          	  ::             :* :*   ** .     :: . :*. *. *****: *** ***

MG06892.1 	TRSGQDLHASFNLLHEDGSPATPSKQKKRTPHGELHFQKT1EEANRTYSTLLRAE~IFEN
NCU01269.1	TRSGQDLQASFSLLHEEGSPATP-RQKRRTPQGELHFQK-~-------------K1LFES
FG01908.1 	TRTGQDLQASFSLLHEDGSPATPAKQKKRTPHGELHFQKT1EEANRTFSHLLRAE~LFES
AN2965.1  	NREGQDLQATYSLLHEDGCPSTPSKTKKRTPHSELHFQKT1EEANRMYSRVLRSE~LFGN
          	.* ****:*::.****:*.*:**:: *:***:.******: ..:.   :    :: :* .

MG06892.1 	SVPQAA-TALSPDQASSRGSNV--HHGTRAQTPPHN---PPPLPASLTPSTPHKNLFSYM
NCU01269.1	TVPQIAPESLSPGHRRASRGYL--HEGTRSQTPPRNGLQAAAAPTTLTPSTPHKNLFSYL
FG01908.1 	SVPQAATPTLSPNQSLPTTSHIPANDGTRAHTPPTNAPAPSLPSSSLTPSTPHKNLFSYM
AN2965.1  	TVPQADLDSLPSNTIRSS----GINDKTRSHTPPSH-VVSALPPASITPSTPHKNLFNYA
          	:***    :*...   .       :. **::*** :   ..  .:::**********.* 

MG06892.1 	SPRHHSSLGGHPTPNRTPQSRHGPNMDTRAEIYSLSPVRFGSQQMLLSPRRQPRAVSKVP
NCU01269.1	SPR-HASLGGHPTPSRTPQSRHGINLDTRAEIYSLSPIKHKSQQLLLSPRKQPRAISKVP
FG01908.1 	SPRHHNQVAGHPTPSKTPQGRHGPNLDTRAEIYSLSPVRFGSQQMLLSPRRQPRAVSKVP
AN2965.1  	SPR---AGSAHPTPSKTPRNQHGPNLNVRSELYSLSPIRYDSQRILETPRKQPRYVNKVP
          	***     ..****.:**:.:** *::.*:*:*****::. **::* :**:*** :.***

MG06892.1 	YKVLDAPDLADDFYLNLVDWGSANVLGVGLGSSVYMWNAQTSRVNKLCTLEDDTVTSVSW
NCU01269.1	FKVLDAPELLDDYYLNLVDWGSANVLGVGLGSSVYMWNAQTSRVNKLCTLEDDTVASVSW
FG01908.1 	YKVLDAPELADDFYLNLVDWGSANILGVGLGSSVYMWNAQTSKVNKLCTLDDDTVTSVSW
AN2965.1  	YKVLDAPDLQDDFYLNLVDWGSSNVLGVGLGNSVYMWNSQTGRVTKLCELKDDTVTSVSW
          	:******:* **:*********:*:******.******:**.:*.*** *.****:****

MG06892.1 	IQK~GTHIAIGTGKGLVQIWDAEKSRRLRTMTGHTARVGSLAWNTHILTSGSRDRLIYHR
NCU01269.1	IQK~GTHLAIGTHKGLVQIWDAEKARRLRTMTGHTGRVGALAWNTHILTSGSRDRLIYHR
FG01908.1 	IQK~GTHLAIGTGKGLVQIWDAEKARRLRTMTGHTARVGSLAWNTHILTSGSRDRLIYHR
AN2965.1  	IQR0GTHLSIGTGKGMVQIWDAERCRRLRTMIGHTNRVGALAWNDHILTSGSRDRHIFHR
          	**: ***::*** **:*******:.****** *** ***:**** ********** *:**

MG06892.1 	DVRAPDQWMRKLVGHKQEVCGLKWNCEDGQLASGGNDNKLMVWDRLSDTPLWKFSDHTAA
NCU01269.1	DVRAPDQWLKKLVGHKQEVCGLKWNCDDGQLASGGNDNKLMVWDKLSDTPLWKYSGHTAA
FG01908.1 	DVRAPDQWLRKLVGHKQEVCGLKWNCEDGQLASGGNDNKLMVWDKLSETPLWKFSDHTAA
AN2965.1  	DVRSPDQYLRRLSGHKQEVCGLRWNTEDGQLASGGNDNKLLVWDKLNETPLYRFSDHTAA
          	***:***::::* *********:** :*************:***:*.:***:::*.****

MG06892.1 	VKAIAWSPHQRGLLASGGGTADRRIIFHDTLRGTVVNEIDTGSQVCNIAWSKNSNEIVST
NCU01269.1	VKAIAWSPHQRGLLASGGGTADRRIIFHDTVRGTVLNEVDTGSQVCNIAWSKNSNEIVST
FG01908.1 	VKAISWSPHQRGLLASGGGTADRRIIFHDTVKGSVINEIDTGSQVCNIAWSKNSNEIVST
AN2965.1  	VKAITWSPHQHHLLASGGGTADRTIKFWNTATGSLIKEVDTGSQVCNLAWSKNSDEIIST
          	****:*****: *********** * * :*  *::::*:********:******:**:**

MG06892.1 	HGYSQNQIVVWKYPSMTQVASLTGHTYRVLYLAMSPDGRVVVTGAGDETLRFWNLFGRRP
NCU01269.1	HGYSQNQIVVWKYPSMTQVASLTGHTYRVLYLAMSPDGKTVVTGAGDETLRFWNLFGKSP
FG01908.1 	HGYSQNQIVVWKYPSMTQVASLTGHTYRVLYLAMSPDGRTIVTGAGDETLRFWSTFGRRP
AN2965.1  	HGYSQNQIVIWKYPRMEQIVSLTGHTFRVLYLAMSPDGQTVVTGAGDETLRFWKIFNRRP
          	*********:**** * *:.******:***********:.:************. *.: *

MG06892.1 	G-AREDNDSG--GRLSDWGIIR
NCU01269.1	RKSVHDLDGGGSGRSGDWGVIR
FG01908.1 	G-SREDGDNG--GRLAELAVIR
AN2965.1  	G--REHGREG--SKLAEWGTIR
          	    ..   *  .: .: . **
```
